# Inositol and PIP2/PIP3 Ratio: At the Crossroad of the Biodynamic Interface Between Cells and Their Microenvironment

**DOI:** 10.3390/biom15030451

**Published:** 2025-03-20

**Authors:** Guglielmo Lentini, Alessandro Querqui, Alessandro Giuliani, Roberto Verna, Mariano Bizzarri

**Affiliations:** 1 Space Biomedicine Laboratory, Department of Experimental Medicine, University Sapienza, 00161 Rome, Italy; alessandro.querqui@uniroma1.it (A.Q.); roberto.verna@fondazione.uniroma1.it (R.V.); 2 Environment and Health Department, Istituto Superiore di Sanità, 00161 Rome, Italy; alessandro.giuliani@iss.it

**Keywords:** myo-inositol, phosphatidylinositol 4,5-bisphosphate (PIP2), phosphatidylinositol 3,4,5-trisphosphate (PIP3), biodynamic interface

## Abstract

Plasma membrane plays a pivotal role in orchestrating motility and invasive processes, as well as mitosis and genome expression. Indeed, specialized regions of the plasma membrane enriched in phosphoinositides—namely PIP2 and PIP3—can accommodate the requirements of the dynamic interface, which mediates the interplay between cells and their microenvironment. The fine-tuned balance between the two phosphoinositides is instrumental in regulating cytoskeleton organization, motility, ion channel activation, and membrane traffic. The balanced expression of PIP2/PIP3 fulfills these functions by activating pathways through several transporter and receptor proteins. These dynamic interactions modulate the interplay with the extracellular environment by decreasing/increasing their exposure on the cell surface. In this way, lipid structures can rapidly either dismiss or recruit specific proteins, eventually favoring their cooperation with membrane receptors and ion channels. Particularly, exposure of proteins can be managed through the internalization of plasma membrane segments, while receptor signaling can be desensitized by their removal from the cell surface. Notably, the equilibrium between PIP2 and PIP3 is largely dependent on inositol availability, as inositol addition enhances PIP2 content while reducing PIP3 via PI3K inhibition. Pharmacological modulation of PIP2/PIP3 balance promises to be an interesting target in different clinical settings.

## 1. Inositol(s), Inositol Phosphates, and Phosphoinositides

Phosphatidylinositol phosphates (PtdIns; phosphoinositides) are phosphorylated inositol phospholipids (PIs) found in eukaryotic cell membranes. They support critical functions, including actin dynamics, membrane trafficking, signal transduction, and regulation of the overall biodynamic interface [1] between cells and their microenvironment.

**Figure 1:**
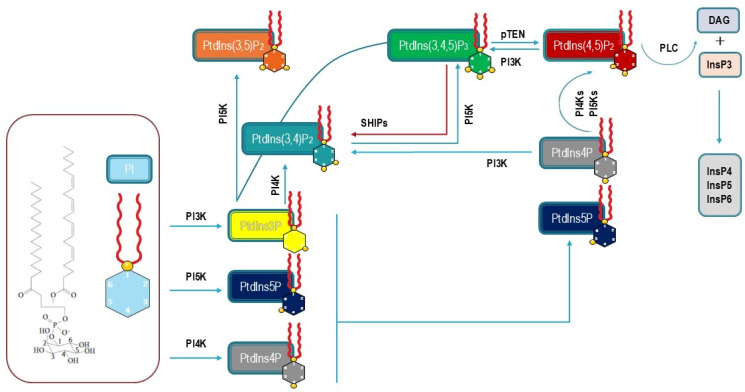
Phosphatidyl-inositol, inositol phosphate, and phosphoinositide synthesis in mammalian cells. The diagram shows the metabolic transformations involving phosphatidyl-inositol as the starting brick under the activity of different phosphatidyl-inositol kinases (PI3K, PI4K, and PI5K), and phosphatases (SHIPs and pTEN). PIP2 is converted into DAG and InsP3 is regulated by PLC. InsP3 is further transformed into inositol-phosphates (InsP4, InsP5, and InsP6).

Phosphoinositides are enriched in stearic and arachidonic acids [2], linked by a glycerol moiety to a water-soluble inositol headgroup as reported in Figure 1.

Inositols belong to the family of cyclitols, which are cycloalkanes with a hydroxyl group on each of at least three or more carbon atoms. They participate in many cellular processes such as water stress, membrane biogenesis, cell wall formation, signal transduction, osmoregulation, ion channel physiology, salt tolerance, and phosphate storage [3]. In plants, cyclitols accumulate in response to various environmental stress conditions and modulate their interaction with the surrounding environment, promoting complex adaptive and self-defense reactions. The safety properties of these molecules—usually known as “compatible solutes”—allow cells to accumulate them to high concentrations and to tolerate large changes in concentration without major adverse effects on cell function.

Inositols are the “bricks” from which both PIs and PtdIns originate. Myo-inositol (myo-Ins)—by far the most abundant inositol isomer present in mammalian cells—is characterized by two hydroxyl groups in the axial position, while the remaining four hydroxyl groups are parallel to the plane of the carbon ring. Eight enantiomeric isoforms of inositol have been identified, as well as their phosphorylated forms. Notably, myo- and scyllo-inositol show a widespread distribution among the different classes of living organisms, including mammals and bacteria. The conversion of myo-Ins into –scyllo and –D-Chiro-Inositol requires the participation of the enzyme epimerase [4,5]. Furthermore, -myo, -scyllo and –D-Chiro-Inositol have been found to enter mammalian cells through active and passive transport processes. Intriguingly, myo-Ins can be autonomously synthesized by both archaea and eukaryotic cells from glucose-6-phosphate. In mammals, exogenous myo-Ins is incorporated into PIs and PtdIns, as mammalian cells—being deficient in myo-inositol kinase (MIK)—are unable to directly phosphorylate myo-Ins. Therefore, the synthesis of InsP depends on the flux of InsP3 produced by the catabolism of phosphatidylinositol 4,5-bisphosphate (PtdIns(4,5)P, or PIP2) catalyzed by phospholipase C (PLC). From InsP3, the concerted action of several inositol kinases produces InsP4, InsP5 and InsP6, with the latter being the most abundant intracellular inositol phosphate [6].

It is worth noting that inositols, InsPs, and PtdIns accomplish this task by managing dynamic transitions between inositol-derived compounds through the involvement of phosphate groups. The phosphate group is a central theme in cell signaling, as it constitutes the currency of exchange in intracellular communications. Phosphate groups released from PtdIns and InsPs under the influence of diverse cues can be likened to “second messengers”, i.e., diffusible molecules that transduce extracellular cues into autocrine/paracrine signals. The cumbersome configuration of phosphate imposes geometric constraints on ligand–protein interplay and contributes its negative charge (at physiological pH), thereby bestowing specificity to its interactions with target proteins. Moreover, inositol displays other appreciable properties that sustain its role as a signaling molecule: it is chemically stable, small, and rapidly diffusible in the cytosol. Furthermore, both exogenous and endogenous sources can modulate intracellular inositol availability in response to an impressive number of stimulations. Therefore, the inositol molecule can be viewed as a “six-bit signaling scaffold” [7], and this can lead to 64 different combinations of one-to-six monoester phosphate groups and 7 PtdIns (Figure 1). Thus, the intrinsic plasticity of inositol makes this molecule an ideal candidate for the synthesis of a plethora of second phosphorylated messengers, including the most recent diphosphoinositol polyphosphates [8]. Conclusively, as suggested by R. Michell, the ability to divert a minor flux of carbon from central energy metabolism toward a chemically stable polar entity with versatile properties should be viewed as a “beneficial evolutionary stratagem” [9].

Visibly, myo-Ins plays very different roles in prokaryotes and eukaryotes. In prokaryotes, myo-Ins is mostly involved in preserving osmolarity and providing basic defenses against host-dependent reactions [10]. In eukaryotic cells, inositol participates in several signaling pathways either as inositol-phosphate or phosphoinositide component, in both the cytosol and cell membranes, in addition to osmolarity control and detoxification activities [3]. Consequently, a reduction in inositol availability can have profound consequences on many biological targets and human diseases [11,12]. Furthermore, inositol depletion negatively affects PtdIns’ homeostasis, by specifically reducing PIP2 content [12], thus highlighting a clear link between inositol deregulation and downstream effects on phosphoinositides.

A global analysis of metabolic network wiring—considering the entire set of sequenced organisms—highlights that the most prominent ‘unique chemical reactions’ differentiating eukaryotes and prokaryotes are linked to inositol metabolism [13]. Notably, all the eukaryotic-only edges involve (directly or indirectly) inositol metabolism. This is a signature of the role of inositol in ‘purely multicellular/eukaryotic’ features like differentiation and cancer. The following chemical reactions are present in eukaryotic metabolic networks, but they are absent in prokaryotic ones: 1-Phosphatidyl-D-myo-inositol/1-Phosphatidyl-1D-myo-inositol 4-phosphate; 1-Phosphatidyl-1D-myo-inositol 4-phosphate/1-Phosphatidyl-D-myo-inositol 4,5-bisphosphate; (GlcNAc)1 (Ino-P)1/GlcN)1 (Ino-P)1; 1-Phosphatidyl-D-myo-inositol/(GlcNAc)1 (Ino-P)1.

All these reactions are involved in the phosphorylation/dephosphorylation of inositols, supporting the role of Ins as “second messengers”. Specifically, the eukaryotic organization makes membranes the preferred site of signalling—the “gates”—between the external environment and the cell, allowing for cell differentiation (and thus, the subsequent development of specialized tissues and organs).

## 2. Phosphoinositides as Gates of the Membrane Biodynamic Interface

Complex systems cannot interact directly, as they need to coordinate and integrate multiple levels of organization. In these systems, sequential causal pathways influence each other and cannot be isolated from the “whole”. Biophysical and chemical cues can exert appreciable effects only through integration into homodynamic processes. This integration requires the interaction between two complex systems and occurs in a ‘shared territory’, i.e., an interface, a dynamic structure whose properties change over time, displaying different, emergent patterns [12]. Consequently, we cannot fully appreciate its characteristics by studying the participating systems as “separate” entities. Both environment and cell are complex systems characterized by multiple stable states emerging; thus, their interaction changes over time. This picture shapes the dynamic interface and necessitates a systems biology approach in performing an appropriate investigation [14].

Usually, mutual interaction among systems is estimated in terms of regression analyses that correlate the input/exposure variance, with the variance of the output (health related) being observable. However, this approach, albeit useful in unveiling statistical associations, fails in assessing true causal relationships. Indeed, the interface may not act as a “cause” in the usual mechanistic sense but may act by constraining and canalizing several environmental cues, therefore assigning a “meaning” to the “signal” that is further conveniently “processed”. In short, a purely regressive approach eliminates the consideration of the state of the two interacting systems that can drastically change the functional form of the relationship and the observed output of the system at hand [15]. Therefore, interfaces establish strict limits on how and what can be “transmitted”.

Interfaces are composed of diverse components, reciprocally entangled to sustain dynamic processes. Yet, the dynamic process supported by these components is the core of the interface. The cytosolic membrane, a dynamic wall with an intertwined array of mechanisms for controlling the interchange of mass and energy, as well as the response to environmental stresses and cues, constitutes the biodynamic interface between the extracellular environment and eukaryotic cells. Membranes envelop distinct subcellular structures that are compartmentalized to allow separation between biochemical reactions with semi-permeable boundaries. In addition, by providing an extensive surface (occupying more than 20 times the cell area) [16], the membrane can host many proteins, cytokines and enzymes, allowing for the functioning of spatially and temporally coordinated dynamic processes. Several proteins—including transmembrane motifs—are permanently included within membranes, whereas others are only transiently associated, depending on the specific stimulation/response required. Therefore, specific regions of these membranes behave as hubs, where cues from the internal/external milieu can be properly managed in order to elicit an integrated response. These organized structures enable synchronized reactions that would otherwise be impossible in a purely diffusive context like ordinary solutions. The overall effect is the compartmentalization of specific enzymatic processes that are constrained in space (volume-confined reactions and surface-confined reactions); such restrictions influence both the rate and the specificity of enzymatic reactions by enhancing/decreasing their kinetics [17].

The membrane fulfills its role in releasing critical factors, selectively activating several pathways, and regulating cell trafficking through several transporter and receptor proteins [18]. These protein complexes are, in turn, allowed to fulfill their effects through the dynamic connection they establish with membrane lipids—specifically cholesterol and inositol-related lipids (PtdIns)—that modulate their interaction with the extracellular environment by decreasing/increasing their exposure on the cell surface. It is worth noting that lipid-based structures can sustain a fine-tuned dynamic equilibrium of embedded protein molecules by changing their location in the membranes, eventually favoring their interactions with membrane receptors and ion channels. In more detail, the exposure of specific protein species can be managed through the internalization of plasma membrane segments, while receptor signaling can be modulated or even abolished by their removal from the cell surface. Increasing evidence points that inositol derivatives play a central role in orchestrating this crosstalk.

These molecular components participate in the architecture of complex lipids that serve as protein-anchoring structures, activators of enzymatic pathways, modulators of ion channels, and as pivots of dynamic gates included in the membranes of the cell and its organelles—Golgi apparatus (GA), endoplasmic reticulum (ER), lysosomes (LYs), nuclear membrane (NM), and endosomes (ES)—that mediate the overall membrane trafficking [19]. The fact that a “simple” molecule, such as inositol (and its derivatives)—only involved in simple and largely non-specific functions in prokaryotes [12])—could have been integrated into the membrane to serve as a “pivot” of the signaling system demonstrates the high plasticity of biological adaptive mechanisms.

Indeed, an alternative use of a simple organic molecule can enact a cascade of events that ultimately favor a transition towards a superior form of organization, making possible a functional compartmentalization that pivots on the selective competency of the biodynamic interface.

Among the inositol derivatives, PtdIns deserve a special attention. Phosphoinositides comprise less than 1% of cell lipids, yet they play very important roles in major signal transduction pathways, serving as docking sites for signaling effectors and as precursors of secondary messengers. In short, they act as critical signaling molecules located at the interface between the extracellular matrix, the cell membrane, and the cytoskeleton [20].

The dynamic properties of PIs and PtdIns emerged in the early 1950s, upon the stimulation of exocrine pancreatic activity (the so-called ‘phospholipid effect’) [21]. The importance of PtdIns in the signaling system anchored to cell membranes also involves the soluble inositol phosphates (InsPs) and inositol pyrophosphates (PP-InsPs), which display an intricate orchestration in response to a wide variety of chemical and biophysical stimuli [22]. In addition to the fundamental role of Ins(1,4,5)P3 (InsP3) in regulating Ca^2+^ intracellular fluxes, InsPs and PP-InsPs participate in several other functions, including gene transcription, micro-RNA (miRNAs) release, nuclear organization, metabolic modulation, and protein phosphorylation, just to mention a few [23,24].

A critical component of the biodynamic interface regulating the interplay between cells and their microenvironment is the fine-tuned equilibrium between two phosphoinositides—phosphatidylinositol 4,5-bisphosphate (PIP2 or PtdIns (4,5)P2) and phosphatidylinositol 3,4,5-trisphosphate (PIP3 or PtdIns (3,4,5)P3), mostly represented on the inner leaflet of the cell membrane. It is worth noting that under the stimulation of different biophysical and chemical cues, rapid bursts of PIP2 and PIP3 synthesis occur, resulting in dramatic changes in the PIP2/PIP3 ratio. Several interacting enzymes carry out these changes [25]. The dephosphorylation of PIP3 into PIP2 is ensured by phosphatases (inositol polyphosphate 5-phosphatase 1; SHIP1) and the Tensin homolog deleted on chromosome 10 (PTEN) phosphatase, which ensures the maintenance of the PIP2/PIP3 homeostasis. Exogenous administration of myo-Ins significantly increases the rate of PI synthesis and PIP2 availability by up-regulating the expression of ISYNA1, the gene encoding for the inositol-3-phosphate synthase enzyme that converts glucose into myo-inositol 1-phosphate [26,27].

The balance between PIP2 and PIP3 contributes to shaping a dynamic gate that compartmentalizes metabolic networks and signaling cascades, bestowing a specific directionality to the transduction of a wide array of biophysical and chemical stimuli. It is worth noting that such a structure is highly sensitive to several environmental influences, including chemical, biophysical, and ionic stimulation. The gate easily undergoes a transition across different conformations that, following changes in the PIP2/PIP3 ratio, induce a cascade of associated events, including the activation of specific inositol phosphates and other signaling molecules [28]. Specifically, PIP2 has emerged as a true signaling intermediate, being regulated by hormones, and it serves as an effector of multiple downstream cytokines and proteins [29].

Conspicuously, those effects act mostly through cytoskeleton (CSK) remodeling [30,31], which is instrumental in activating the response of membrane receptors, including HER2, RTKs, IRS, GPCR, EGFR, and ion channels (TRPM and KCNQ1) [32]. Furthermore, changes in PIP2 can modify the bilayer properties of membranes, thus altering the pattern and function of proteins embedded in the membrane leaflet [33]. Perturbations of the delicate balance between PIP2 and PIP3 levels hence result in a few alterations in ion channels, neurotransmitter signal transduction, synaptic plasticity, and cytoskeletal remodeling [34,35], finally leading to aberrant morphogenesis and degenerative diseases [36,37]. It is worth noting that changes in the PIP2/PIP3 ratio are instrumental in modulating actin–myosin contractility and plasma membrane expansion during tissue morphogenesis [38], and, as such, they play a critical role in the modulation of cell motility and mitosis. PtdIns are key drivers in multiple temporally defined membrane remodeling steps involved during events of mitosis—including cell rounding, spindle orientation, cytokinesis, and abscission [31]. PIPs may also regulate membrane curvature due to the geometric hindrance provided by their large headgroups and asymmetrical distribution between inner and outer leaflets, even independently of proteins [39].

Moreover, in the nucleus, changes in the phosphoinositide content involving the PIP2/PIP3 ratio occur shortly after DNA damage and play a critical role in response to genotoxic stresses [40]. The phosphoinositide content in the nuclear membrane changes during cell cycle, differentiating process, tissue repair, and in response to stressors and growth factor stimulation [41,42]. These data suggest that the PIP2/PIP3 ratio constitutes a new signaling interface for DNA repair pathway selection.

## 3. Synthesis and Distribution of PIP2 and PIP3

### 3.1. Synthesis of PIP2

Synthesis of phosphatidyl-inositol phosphates occurs in the endoplasmic reticulum (ER) through a two-step process, starting with the conversion of phosphatidic acid (PA) into cytidine diphosphate (CDP-DAG), before the final conversion into PIs under the catalytic activity of phosphatidyl-inositol synthase (PIS). The first step is fostered by CDP-DAG synthase (CDS1 and 2) enzymes and requires the participation of the 1L-myo-inositol 1-phosphate cytidylyl-transferase enzyme (CTP), while inositol is added in the second step. PA is provided by de novo synthesis or from the recycling of PtdIns(4,5)P2 under the activity of PLC. PLC activation splits the phosphoinositide into diacylglycerol (DAG) and inositol 1,4,5-trisphosphate (InP3). While InsP3 serves as a second messenger, DAG is rapidly converted into PA to restart PI synthesis [43]. Remarkably, due to the compartmentalization of inositol metabolism, PIs and PA translocate from the ER to the plasma membrane by taking advantage of a cluster of specific PI transfer proteins (PITPs).

Phosphatidylinositol 4,5-bisphosphate is mostly synthesized from PI at the plasma membrane under the phosphorylating activity of PI 4-kinase (PI4K) and PI4P5K. Three different PI4K isoforms are currently known, and PI4K Type IIIα (PI4KIIIα) plays a major role in the synthesis of PI4P. Further transformation of PI4P into PIP2 is catalyzed by PI4P 5-kinases (represented by three isoforms). PI(4,5)P2 is hydrolyzed by PLC, which releases DAG and InsP3, both of which act as second messengers. InsP3 binds to specific receptors located at the ER surface where it promotes the release of Ca^2+^ into the cytosol. Moreover, InsP3 is a substrate for the synthesis of inositol phosphates (InsP4, InsP5, and InsP6) and pyro-phosphates (IP7 and IP8), which play a relevant role as signaling molecules in several critical pathways [23]. DAG, retained at the plasma membrane, is an activator of protein kinase C (PKC), fostering PKC translocation from the cytosol to the plasma membrane. DAG is also involved in regulating Ras-GRP4 (Ras guanyl-releasing protein 4) and reconstituting PA stores. Notably, PA participates in pyrophosphate signaling (namely in association with IP6K1), recruiting PIP5K, and modulating membrane trafficking events by shaping membrane topology [44].

### 3.2. Intracellular Localization

**Figure 2:**
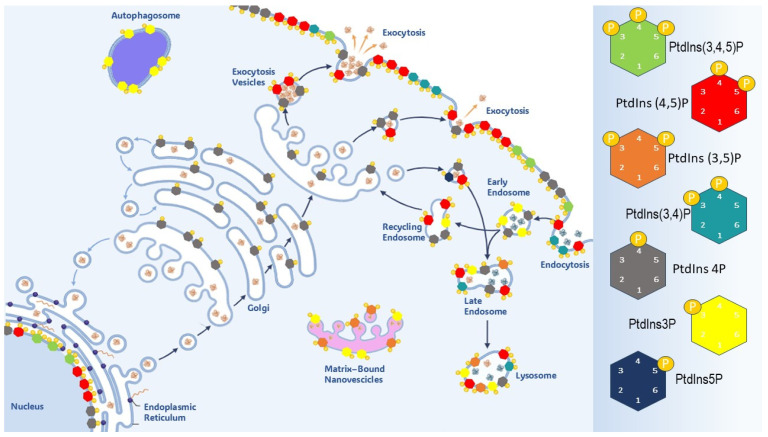
PtdIns’ selective distribution in the mammalian cell. Phosphoinositides are specifically represented on the surface of membrane of the Golgi apparatus, nuclear membrane, endoplasmic reticulum, autophagosomes, lysosomes, and endosomes.

Asymmetry in PtdIns’ distribution (including different localization of PIs and related enzymes) plays specific roles in regulating endosomal trafficking pathways, across various membrane compartments (Figure 2). This asymmetry is instrumental in ensuring the internalization/externalization of membrane components, and, thereby, in regulating the release of cytokines and endocrine factors at the cell surface [45].

Furthermore, this asymmetry is essential to preserve polarity and identity in epithelial cells, where apical and basolateral membranes display different structural and functional properties. These differences are critical for the overall tissue architecture homeostasis and tissue/organ development and have been extensively investigated in many cell types, including neurons [46]. The seminal work of Gassama-Diagne et al. [47] demonstrated that PIP3 is usually confined to the basolateral membrane of polarized epithelial cells (canine kidney MDCK cells), while the apical surface is enriched in PIP2. However, the addition of PIP3 induced a profound transformation of the apical region due to the recruitment of PI3K at the cell surface and the concomitant exclusion and reduction of PIP2 and other proteins. Conversely, treatment with PI3K inhibitors reversed this distribution, restoring normal polarity.

PIP2 has been found to be highly enriched within segregated domains, with a very limited size (~73 nm) [48], and, notably, its concentration exceeds that of PIP3 by 6–2 times [49]. PIP2 is the most abundant among all seven species of PIPs, and it is instrumental in bestowing electrostatic properties to membranes, allowing for their proper orientation, which is needed for lipid signaling events to occur [50]. Although preliminary immunofluorescence studies have identified only minimal intracellular localization of PIP2, recent innovation in immunofluorescence detection highlighted that PIP2 is concentrated in high amounts within specific structures, such as endosomes, the endoplasmic reticulum (ER), the Golgi complex, mitochondria, and the nucleus. Moreover, in the same locations, the presence of both phosphatidyl-kinases (PIPKs) and phosphatidyl-phosphatases is well documented [51].

PtdIns are concentrated at the cytosolic surface of membranes, whereas synthesis of PI, the precursor of phosphoinositides, occurs in the ER. Phospholipids then shuttle between cell and organelle membranes either by vesicular transport or via specific transfer proteins. The inositol headgroup of PtdIns can be reversibly phosphorylated at three positions, 3, 4 and 5, thus leading to seven isoforms, PtdIns(3)P, PtdIns(4)P, and PtdIns(5)P); three bisphosphorylated isoforms (PtdIns(3,4)P2, PtdIns(3,5)P2 and PtdIns(4,5)P2); and one trisphosphorylated (PtdIns(3,4,5)P3) isoform. Phosphorylation of inositol thus results in the production seven PtdIns species, each one with a specific intracellular distribution. Phosphoinositides are predominantly located on the interior face of membranes, including the nuclear membrane. PI 4-phosphates—including phosphatidylinositol 4-phosphate (Ptdins4P) and PIP2—are mostly located along the exocytic pathway, at the cell, and the nucleus. On the contrary, PI 3-phosphates—phosphatidylinositol 3-phosphate (PI(3)P), phosphatidylinositol 3,4-bisphosphate (PI(3,4)P2), phosphatidylinositol 3,5-bisphosphate (PI(3,5)P2), and PIP3—are synthetized mostly at the cell surface, but they translocate to the endosomal system. Remarkably, PIP2 and PIP3 are mainly concentrated in the leaflet of the plasma membrane and excluded from endosomes, while PI(3)P is synthesized in the latter compartment and is absent from the plasma membrane. This asymmetric distribution is consistent with the distinct physiological function of each class of PIPs. PIP3 sustains exocytosis-related processes, while PIP2 participates in endocytosis, given its competence in anchoring several coat and cytoskeleton proteins to the cytosol membrane. A paradigmatic example evidencing the critical role played by PIP3 in exocytosis is the insulin-mediated exocytosis of GLUT4 transporter proteins. In adipocytes, SHIP2 overexpression attenuates the concentration of GLUT4, while promoting PI(3,4)P accumulation due to the dephosphorylation of PIP3. Conversely, silencing of SHIP2 restores PIP levels and enhances insulin sensitivity [52,53].

Notably, intense stimulation—supported by hormones and cytokines—can lead to asymmetric distribution of PIP3 versus PIP2. Insulin determines extensive membrane ruffling and localization of PIP3 on GRP1 PH domain to the ruffles [54]. Growth factors, such as PDGF and RTKs, enhance the activation of PI3K and promote membrane ruffling, whereas PI3K inhibitors antagonize this effect [55]. Asymmetric spreading of PI3K is also observed during cell migration and invasiveness. PI3K concentrates at the leading front during neutrophil chemotaxis, while at the cell rear, PI3K is virtually absent. PI3K accumulates at the leading membrane, while PIP2 and PTEN are concentrated at the rear of motile cells [56]. The migrating-invasive process requires tight cooperativity with some CSK components, including actin. Indeed, PI3K accumulation within the leading front is disrupted when actin remodeling is inhibited by latrunculin or jasplakinolide, toxins that inhibit actin polymerization or depolymerization, respectively [57]. Therefore, fluctuations in PIP3 concentration, alongside orchestrated changes in the CSK, ensure cell polarity and chemotaxis in response to different cues. Overall, accumulated data suggest that localized synthesis of PIP3—with a resulting increased concentration—occurs in active zones of the cell surface associated with membrane protrusion and cell motility. On the contrary, PIP2 enhances cell-substrate anchorage and provides a specific directionality in cell trajectories [58].

Notably, there is high correlation between the localization of high PIP3 concentrations in ruffles and the localization of exocytosis at these sites, where transferrin and LDL receptors from the endosomal system accumulate in membrane ruffles upon the orchestrated activity of EGF and PIP3 activation [59]. Interestingly, inhibition of PIP3 synthesis interrupts membrane ruffling, suggesting that PIP3 is mandatory for ensuring exocytosis. A further mechanism through which PIP3 regulates membrane insertion involves some protein kinases downstream of PDK1 [60], including Akt [61], three SGK isoforms, and PKC [62].

Similarly, PIP2 displays an asymmetric distribution within cell membranes. Furthermore, it is even more intriguing that the immediate PIP2 precursor—PI(4)P—is virtually absent from the plasma membrane while being concentrated in the Golgi apparatus and other intracellular membranes, where PI(4)P associates with several PI 4-kinase enzymes [63]. However, PI 4-kinase has been found in plasma membrane of mammals [64], suggesting that PI(4)P may derive from the Golgi. In this way, appreciable PI(4)P levels may concentrate on the plasma membrane, thereby providing enough precursors to enable PIP2 synthesis.

PIP2 was shown to accumulate preferentially on the leaflet of cytosol membrane—in proximity to actin and ruffles—rather than at the intracellular level, with the notable exception of nuclear PIP2 [65]. Remarkably, PIP2 establishes a functional relationship with N-WASP family proteins, as well as with several molecular factors—including cofilin, profilin, and vinculin—that participate in actin polymerization [56]. However, PIP2 levels rapidly change their location on the cytosol membrane in response to activated PLC [66] and several growth factors, like EGF [67]. Overall, these data indicate that PIP2 turnover in the cytosol membrane can display either an increase or decrease at localized regions in response to physiological and pathological cues.

The segregation of different classes of PtdIns across membranes belonging to diverse structural components (cytosol membrane, organelles, and nucleus) imposes a vector directionality to membrane traffic, bestowing selectivity in the transport system from one compartment to another. In this way, PIPs can act like zip codes or signature motifs of those membranes. Accordingly, signal recognition occurs through a coincidence detection mechanism, whereby interaction affinity increases progressively due to subsequent cooperativity enacted by associations with additional components (lipids and proteins).

Particularly, in the nucleus—together with phosphatidylinositol phosphate kinase type 1 (PIP5K1A)—PIP2 is required for the stabilization of p53 [56]. Nuclear PIPKI-α binds to p53 upon stress, resulting in the production and subsequent association of PIP2 with p53, which subsequently promotes the interaction between p53 and the small heat shock protein HSP27. Conversely, inhibition of PIP2 leads to p53 destabilization. Furthermore, PIP2 binds to the nuclear steroidogenic factor 1 (SF-1), which is critical for folliculogenesis and aromatase expression in ovarian granulosa cells [68]. Intriguingly, SF-1 activity dramatically increases downstream of enzymatic PIP2-phosporylation mediated by inositol polyphosphate multi-kinase (IPMK). It is worth mentioning that IPMK can regulate transcriptional events by modifying the protein–lipid cargo [69], removing the inhibitory effect of PIP2 on SF-1 transcription. However, investigations regarding the role of PIP2 in nuclear events tied to the regulation of gene expression are still in their infancy and remain elusive, partly because few nuclear effector proteins have been identified [70].

**Figure 3:**
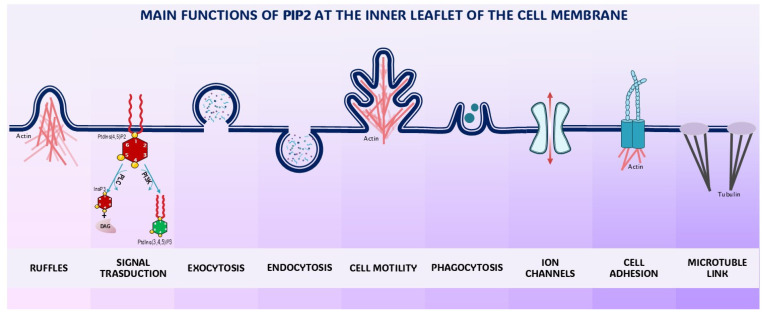
Schematic diagram illustrating some processes regulated by PIP2 at the plasma membrane. PIP2 plays a critical role during endocytosis/exocytosis processes, in supporting motility and adhesion (namely by reinforcing cell connection to the substrate), through modulation of microtubules and other cytoskeleton components. See text for explanations.

In summary, PIP2 and PIP3 are integral constituents of the interface mediating the interplay between cells and their microenvironment, specifically involved in managing the endocytic and exocytic machinery (Figure 3) [71]. Some excellent reviews have recently addressed those issues [72,73].

### 3.3. The Pivotal Role of PI3K

PtdIns phosphorylated in position −3 is produced by eight different PI 3-kinases (PI3Ks), grouped into three classes based on their association with regulatory subunits and preferred substrates. Class I PI3Ks mainly produce PI(3,4,5)P3, class II generates PI(3,4)P2 and PI(3)P, whereas class III solely forms PI(3)P. Even more important is how PI3Ks are partitioned based on their substrate specificity [74]. Class I PI3Ks are involved in mediating enzymatic reactions downstream of plasma membrane-bound receptors, whereas class II and III PI3Ks primarily regulate vesicular trafficking and intracellular processes, particularly those occurring within the endosome/lysosome system [75].

Phosphoinositide 3-kinase (PI3K) specifically phosphorylates the 3-hydroxyl group of the inositol ring in phosphatidylinositol lipids, enabling them to become ligands for several proteins. PI3Ks include three clusters (Classes I, II and III) of enzymes, differing in their respective substrate specificity. Namely, Class I PI3Ks selectively phosphorylate PI(4,5)P2. It is worth noting that a broad range of plasma membrane-bound receptors can convey different signals to PI3Ks, ultimately activating the PI3K-dependent cascade. PI3K enzymes are heterodimers composed of a p110 catalytic subunit (containing the kinase domain) and a regulatory subunit, which retains the molecule in an inactive condition. Five regulatory subunits (p85α, p85β, p55α, p50α, and p55γ) have been recognized so far, while four catalytic subunits (p110α, p110β, p110γ, and p110δ) are present in mammalian cells. Notably, many domains—present in catalytic and regulatory subunits of different PI3K isoforms—may significantly affect kinase activity. These structures include the RAS-binding domain (interacting with the Ras and Rho families), SH2 domains (binding to the pYXXM motifs of phospho-tyrosine residues), and regions associated with the βγ subunits of heterotrimeric G proteins. Interestingly, different inputs interact through specific receptors with different PI3K isoforms, adding further complexity to the overall regulation of this critical hub of phosphorylating enzymes [76]. It is worth mentioning that p110α heterodimers play a critical role in transducing insulin signaling, ensuring glucose metabolism [77], supproting immune functions [78], and contributing to cancer transformation; they are among the most frequently deregulated or mutated genes in several tumors [79].

The immediate product downstream of the activation of PI3K—its distinctive mark—is the synthesis of phosphatidylinositol 3,4,5-trisphosphate (PI(3,4,5)P) from PI(4,5)P2, the preferred substrate for PI3K. The synthesis of PI(3,4,5)P3 is tightly connected to the subsequent activation of several key molecular factors. Notably, both PI(3,4,5)P3 and PI(3,4)P2—resulting from the dephosphorylation of PI(3,4,5)P3 upon the activation of the SHIP family of phosphatases (Src homology,SH2) containing inositol polyphosphate 5-phosphatase—are instrumental in recruiting and anchoring several effector proteins to the membrane. Here, the most relevant factor is the availability of phosphoinositides phosphorylated in position 3 (PI-3) [19]. Experimental manipulation of the intracellular pool of PI-3 showed that increased availability of PI(3,4,5)P3, PI(3,4)P2 and PI3P is required for the transduction of the receptor tyrosine kinase signaling [80] and modulation of clathrin-mediated endocytosis (CME) [81].

The dynamic interplay between PI3K and receptor tyrosine kinases (RTKs) and G-protein-coupled receptors (GPCRs) plays a pivotal role in regulating the cell/tissue PIP3 availability, given that PI3K activity is induced upon stimulation by activated RTKs and GPCRs [76]. Thus, PI3K synthesis and activation (mostly though the p110β subunit) are mandatory for transducing metabolic and morphogenetic processes mediated by RKs and GPCRs [82,83].

The specific localization of PI-3-phosphates, and the reversible character of phosphorylation make these compounds key regulators of the PIP2/PIP3 balance, influenced by intra- and extracellular stimuli from both chemical and biophysical stressors. PtdIns exert their function at membranes via the recruitment of effector proteins containing PtdIn-binding domains. Protein interact with PtdIns through either unstructured regions—as happens with profilin and other actin-related regulatory proteins [84]—or specific modules, such as the pleckstrin homology domain [85]. Phosphoinositides achieve direct signaling effects through the binding of their headgroups to the cytosolic domains of membrane proteins. In this way, PtdIns can modulate protein functions, namely by recruiting other proteins that in turn dynamically interact with cytoskeleton components.

This architectural system integrates protein domains that selectively bind to PIs and PtdIns, which are specific to each subcellular organelle and plasma membrane compartment. These domains mediate the attachment of proteins to membranes—according to a continuous dynamic assembly/disassembly regimen—that is required to either trigger or inactivate signaling pathways. In eukaryotes, those structures are regrouped into super-families—including FYVE, PH, and PX domains—which recognize membrane lipids through a PI-code that is becoming more apparent due to research over the last twenty years [73]. This body of evidence calls for a reappraisal of the classic notion of the cell membrane—introduced by Singer and Nicholson in the 1970s [86]—and posits that membranes are dynamic structural arrays organized into distinct functional areas, occurring through the establishment of a protein–lipid code [87]. The integrated framework of PtdIns/PIs and proteins within selected regions of the plasma membrane behaves like a “continuous group”, capable of performing specific tasks and ensuring a proper, dynamic gate between the cell and its microenvironment. Any chemical, ionic, or physical stimulus must interact with these structures in order to be converted into a proper intracellular signal. These dynamic regions establish strict relationships with cytoskeleton components [88]. These associations are required for ensuring gate stabilization as well as bestowing specific functions, such as the formation of lamellipodia, filopodia, podosomes, and invadosomes [89]. The stability, localization, and duration of these functional areas are tightly regulated by a plethora of PtdIn-modifying factors, including specific PI-kinases, phosphatases, and lipases. Deregulation of the structural composition of those areas has been linked to several diseases, highlighting their potential usefulness as therapeutic targets [90].

Within these structures, PIP2 and PIP3 play a very relevant role. Indeed, these two PtdIns and their related enzymes—including phosphatidylinositol-4-phosphate 5-kinase (PI4K) and phosphatidylinositol 3-kinase (PI3K)—have been shown to participate in cytoskeletal remodeling and in the biochemical transduction of physical forces, acting as critical components of the biodynamic interface that mediates the dynamic relationships between cells and their microenvironments [91]. Furthermore, a direct interaction of phosphoinositides with mechanically sensitive membrane-bound ion channels in response to mechanical stimuli has recently attracted attention [92].

## 4. Phosphoinositides: The Cytoskeleton, Ion Channels, and Mechanotransduction

A biodynamic interface should manage a wide array of different signals, including those provided by biophysical cues (mechanical, bioelectric, gravitational, and magnetic) and chemical factors (chemokines, growth factors, and hormones) resulting from the interaction with the microenvironment (stroma and ECM) and surrounding cells. It is worth noting that PtdIns are involved in all those processes. Phosphoinositides (in association with InsPs) transduce these stimuli into intracellular chemical signals downstream of PtdIns’ interaction. The initial cue is appropriately processed before ensuring its transduction and can eventually trigger numerous, distinct pathways.

PtdIns modulate a tremendous breadth of horizontal and vertical cell signaling crosstalk spanning the cell membrane and cytoplasm, respectively. Within these regions, high-affinity interactions occur among pleckstrin homology (PH) domain-containing membrane-based and cytosolic effector proteins, including activated protein kinase B (PKB or Akt), protein kinase C (PKC), PLC, and 3-phosphoinositide-dependent protein kinase-1 (PDK1), just to mention a few [93]. Activated PtdIn-related signaling mediates enzymatic organic modification of secondary messenger proteins, given that PIPs are crucial scaffolds, which enable the recruitment of several effectors to perform specific functions, under the constraint of field-dependent forces. These biophysical forces (stiffness, tensile stretching, gravity, and shear stress) exert their effects through mechanotransduction [93]. Mechanical cues are hence converted into intracellular biochemical signals, fostered by the concomitant modification of cytoskeleton (CSK) and nuclear nucleoskeleton (NSK) architecture.

PtdIns play a critical role in transmitting those cues to the CSK, while establishing cooperative interactions with numerous structures and proteins that regulate cell-to-cell adhesion (E-Cadherin and β-Catenin), cell-substrate crosstalk (FAK, integrins, Talin, Src, Vinculin, etc.) and actin remodeling (by alternatively promoting actin polymerization or remodeling), the emergence of stress fibers, and motility/invasion-related structures (filopodia, pseudopodia, etc.) [94]. As Talin’s interaction with PIP2 promotes its binding to the cytoplasmic domain of the β1 integrin subunit, the depletion of PIP2 from focal adhesions temporally separates integrin–ligand binding from integrin–actin force coupling [95]. In turn, integrin-mediated adhesion promotes PIP2 synthesis [96]. Reinforcement of adhesion is therefore instrumental in enhancing PIP2 expression.

Notably, PIP2 binds to and affects actin-binding proteins, such as myristoylated alanine-rich C kinase substrate (MARCKS) [97], cofilin, gelsolin, α-actinin, Wiskott–Aldrich syndrome protein (WASP), and the Rho family of small GTPases (Rho-A) [98]. Moreover, PtdIns modulate motor proteins (ROCK1/2) by interacting with the Rho-A dependent cascade of effectors. Alterations in cytoskeletal organization, specifically actin filament dynamics, can result in gene expression and cell proliferation modifications with the subsequent modulation of intracellular biochemical responses and cellular functions. PtdIns are pivotal actors of cytoskeletal reorganizational events, including vesicle trafficking, cell migration, phagocytosis, and membrane cytoskeletal adhesion, and in this way, they actively participate in phenotypic transition and morphogenetic processes, like the Epithelial–Mesenchymal Transition (EMT). Conversely, manipulation of PtdIns through pharmacological intervention or modification of the cell microenvironment has been demonstrated to be instrumental in inhibiting cancer malignancy and even in promoting a phenotypic reversion/reprogramming toward a non-cancerous phenotype [35].

The stress provided by a mechanical stimulus provokes a profound remodeling of PIP2/PIP3 balance, with a concomitant change in many other factors downstream of PIP2/PIP3. This process initially involves the activation of PI3K, which phosphorylates PIP2 into PIP3. In turn, PIP3 recruits PDK1 to the plasma membrane. This step is critical in the PI3K-elicited signaling cascade, as PDK1 is usually in an inactive conformation within the cytosol. PIP3 removes the autoinhibition exerted by the PH domain of PDK1 and allows for the auto-phosphorylation of the PDK1-kinase domains, a process that is unlikely to occur in the absence of PIP3. Interestingly, a threshold PIP3 concentration is mandatory for PDK1 activation, thus suggesting that the complete blockade of PI3K and PIP3 is not required to fulfill a significant biological effect, as even a limited reduction can be effective [99].

Activation of PDK1 is mandatory to phosphorylate and activate Akt at the threonine residue 308. Together, Akt and PI3K create a unique signaling pathway (Akt/PI3K) that is instrumental in cell mechanotransduction. Moreover, the Akt/PI3K signaling pathway regulates intracellular and extracellular activities in response to mechanical stress and molecular effectors, leading to a robust cellular mechanotransduction signaling cascade. In response to mechanical stress, PI3Ka translocates to the plasma membrane to convert PIP2 to PIP3, which subsequently recruits gelsolin to the plasma membrane. Concomitantly, reduced levels of PIP2 favor the release of cofilin (usually linked to PIP2 beneath the cell membrane) into the cytoplasm, where it promotes actin remodeling and the acquisition of an invasive phenotype. This process is further enhanced by the negative regulation of gelsolin through increased PI3K activity [100].

The involvement of other factors—namely Vinculin increase, disruption of E-Cadherin/β-Catenin junctions, and modulation of ROCK and RhoA complexes—inhibits the Hippo pathway when YAP/TAZ are excluded from the nucleus and migrate to the cytosol. Yes-associated protein 1 (YAP) and transcriptional co-activator with the PDZ-binding motif (TAZ) are downstream transcriptional activators of the Hippo pathway. These effectors are regulated by mechanical cues, specifically matrix stiffness, stretch, and cell density, which influence cell proliferation and differentiation and constitute the ultimate factors participating in the mechanotransduction processes, starting from the membrane and culminating within the nucleus. It is worth noting that modulation of the intracellular pool of phosphoinositides through the addition of myo-inositol—the basic brick precursor of PIPs—can efficiently reverse the cancerous phenotype and antagonize the migrating/invasive properties of cancer cells, specifically by targeting several EMT parameters. Evidence suggests that the modulation of PIP2/PIP3 balance plays a critical role in all these processes, given that the addition of exogenous myo-Ins dramatically increases the abundance of PIP2 [101].

**Figure 4:**
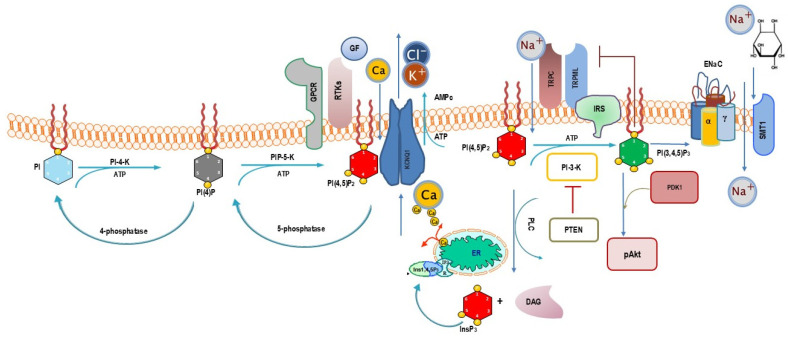
The diagram shows the participation of PIP2 as a central regulator of a number of receptors for chemical ligands, epithelial sodium channels, ion exchangers, voltage-gated potassium channels, and calcium channels and pumps. TRPC1/3/6: transient receptor potential canonical channels 1, 3, and 6; TRPML: transient receptor potential ion channels; GF: growth factors; KCNQ2/KCNQ3: neuronal hetero-tetrametric potassium channels; GPCR: G-protein-coupled receptors; RTKs: receptor tyrosine kinases; ENaC: epithelial sodium channels; SMT1: sodium-myo-inositol transporter-1.

PIP2 has also recently emerged as a central regulator of several receptors for chemical ligands, epithelial sodium channels, ion exchangers, voltage-gated potassium channels, and calcium channels and pumps [102]. Notably, these structures display remarkable sensitivity to biophysical stresses [103]. Usually, ion channels within the plasma membrane interact with local membrane phosphoinositide phospholipids for proper function. We now know that phosphoinositides regulate a large number of ion channels, albeit some channels may not be sensitive to phosphoinositides at all. Other ion channels have an obligate requirement for one species of phosphoinositide, whereas others may accept a broader range of phosphoinositides (Figure 4) [104].

All integral membrane proteins are in intimate contact with a sea of lipids. Thus, it is not surprising that many ion channels evolved to interact with and recognize the distinctive high negative charge and headgroup geometries of polyphosphoinositides. Some studies show crystal structures of PIP2 associated with members of the inwardly rectifying potassium channel family: Kir2.2 and G-protein-coupled inwardly rectifying potassium channel GIRK2 (Kir3.2). Both plasma membrane channels require PIP2 to be active. The structures show specific ion channel–lipid interactions and conformational changes that suggest channel activation when the lipid binds. Moreover, plasma membrane store-operated channels (SOCs)—particularly transient receptor potential canonical channels 1, 3, and 6 (TRPC1/3/6)—and calcium release-activated calcium (CRAC), are activated downstream of PLC activation [105]. PLC cleaves PIP2 into diacylglycerol (DAG) and inositol 1,4,5 trisphosphate (InsP3). While DAG remains bound to the leaflet of the cell membrane, InsP3 migrates into the cytosol and interacts with specific receptors on the endoplasmic reticulum, fostering the release of Ca^2+^ from intracellular stores [106].

Stimulation of SOCs modulates Ca^+^ influx pathways and subsequently regulates vascular smooth muscle cell (VSMC) contraction, proliferation, and migration. In these cells, both PKC and PIP2 play a relevant role in the activation of the transient receptor potential canonical channel 1 (TRPC1). Upon depletion of Ca stores, TRPC1 is phosphorylated by PKC, which itself is stimulated by the PLC-PIP2-DAG pathway thus establishing a potentially direct link between TRPC1 and PIP2. Additionally, TRPC1 is known to be an essential component of various mechanotransduction pathways [107]. In addition to TRPC, the Orai (CRAC) channel—also belonging to the SOC group—is activated downstream of the activation of PLC and the subsequent PIP2 depletion.

For KV channels, the clearest examples of regulation by PIP2 are the KV7 channel family. These are slowly activating channels, some of which are already open at rest. It is widely accepted that for the common neuronal KCNQ2/KCNQ3 hetero-tetrametric potassium channels, PIP2 is essential for their voltage-gated activity, and that depletion of PIP2 renders them inactive, causing the neuron to become transiently more excitable until the lipid is re-synthesized [108].

A direct involvement of ion channels in processes triggered by mechanotransduction has only recently emerged. It is noteworthy that the involvement of mechanosensitive (MS) ion channels was discovered during studies dealing with the mechanical regulation of stem cell activity [109]. Since osmolarity stress on the lipid membrane was probably one of the first vital signals faced by early life forms in water, the presence of MSCs in most cell types appears necessary. However, in multicellular organisms, roles for MSCs have been identified only in excitable cells [110]. This apparent contradiction was solved by the discovery in 2010 of Piezo, an MS channel broadly expressed in many different tissues [109]. Piezo is one of the most sensitive MSCs discovered to date and can be directly activated in the presence of membrane tension without any additional components. Therefore, Piezo appears to be an MSC that primarily responds to mechanical stresses in vivo. The mechanosensitive function of Piezo greatly facilitates the interpretation of loss-of-function studies, favoring a more realistic interpretation of mechano-biology. Notably, it has been shown that Piezo mechano-sensitivity with respect to membrane tension is regulated by PIP2 levels. Activation of PLC and the subsequent depletion of PIP2 subsequently inhibits Piezo mechanosensitive activity [111]. Furthermore, it is worth noting that the participation of both phosphoinositides and ion channels in cancer biology has been documented in several tumors, including breast, colon, and ovarian neoplasms [112].

## 5. Conclusions

**Figure 5:**
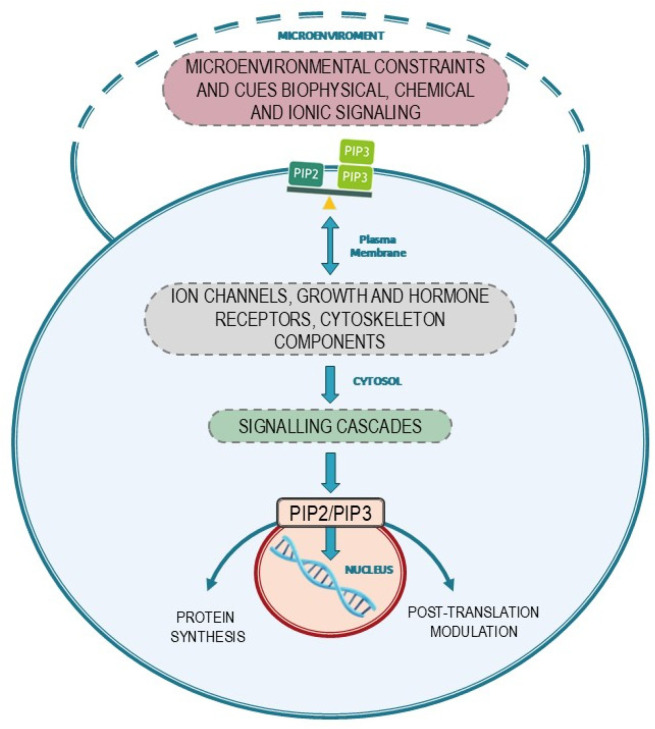
The biodynamic interface: pivotal role of the PIP2/PIP3 balance in transducing environmental stimuli to both cytosol and nucleus.

Available data indicate that processes occurring at the membrane are critical in enacting several transition processes and in processing biological inputs. The plasma membrane plays a pivotal role in orchestrating motility and invasive processes, as well as mitosis and genome expression. Indeed, specialized regions of the plasma membrane can adapt to the requirement of a proper “dynamic interface”, defining the boundaries and mediating the interplay between cells and their microenvironment in both space and time dimensions. Furthermore, the membrane serves as a platform for assembling and integrating countless components, which actively participate in all aspects of the motility process, including force generation, adhesion, and movement regulation (Figure 5).

It is crucial to stress that most of these functions chiefly depend on the delicate balance between PIP2 and PIP3, the two main phosphoinositides that localize beneath the cell membrane, where they regulate cytoskeleton organization, motility, ion channel activation, and membrane traffic. An exemplary proof of principle has recently been provided by studies investigating the transition from a neoplastic to a non-metastatic phenotype. Treatment of MDA-MB-231 breast cancer cells with fish embryonic extracts suppresses the migratory/invasive phenotype. Interestingly, this effect occurs downstream of PI3K inhibition, once the right PIP2/PIP3 ratio has been restored. After treatment, PIP2 distribution beneath the cell membrane increases steadily, and consequently, cofilin remains close to PIP2, while in untreated cells, cofilin spreads throughout the cytosol. Cofilin dispersal in cytoplasm is instrumental in activating cytoskeleton remodeling, severing F-actin filaments, and shaping a motile phenotype. Conversely, a decrease in PIP2 and an increase in PIP3 levels enhance the release of cofilin and promote cell invasiveness.

It is worth noting that all the above-mentioned processes involve symmetry breaking, assigning different functions to alternative locations in space. These alternative locations, in most cases, exhibit a binary character (e.g., inner/outer membrane). The same binary character is also evident in the concentration balance of two alternative molecular species. This binary character makes it possible to link space and time through a digital form of control very that is common in both artificial and natural systems: the so-called ‘toggle-switch’.

The most common example of a toggle-switch is a bi-stable circuit, made of two mutually repressing elements, A and B, which impose two different attractor states on the circuit: (1) A is expressed at its typical level, while B is silenced, and (2) A is silenced, while B is expressed at its typical level [113]. In addition to this simple model, a bi-stable regulation can also emerge from reaching a threshold level of a micro-environmental signal. The most consolidated example of this type of regulation is the allosteric effect that causes hemoglobin to shift back and forth between the R and T states based on the partial pressure of oxygen [114]. In the case of gene expression, this bi-stable dynamic can, in principle, affect any kind of gene through multiple ways [115]. Bi-stable switches allow for a more reliable control of the system compared to other control strategies. The paradigmatic biological example is the λ-phage switch in E. coli. The switch initially remains in the dormant (lysogenic) state but can be flipped into the active (lytic) state due to the presence of the bacterial protein RecA. This induction event occurs when the cell starts to produce RecA to repair DNA damage because of, e.g., a burst of UV light. Such a digital control is not only highly resistant to noise but also allows for a dynamic alternation of discrete states (e.g., open/closed states of a gate) of the biodynamic interface that governs the mutual adaptation of the microenvironment and cell states.

Conclusively, mounting evidence shows that the PIP2/PIP3 ratio plays a role in shaping the cell biodynamic interface, acting like a “toggle-switch”. The modulation of the above-mentioned relationship can have profound effects on cell biology and physiology by modulating the transduction of several different chemical and biophysical factors.

## References

[1] Shewan A., Eastburn D.J., Mostov K. (2011). Phosphoinositides in Cell Architecture. Cold Spring Harb. Perspect. Biol..

[2] Kim Y.J., Sengupta N., Sohn M., Mandal A., Pemberton J.G., Choi U., Balla T. (2022). Metabolic routing maintains the unique fatty acid composition of phosphoinositides. EMBO Rep..

[3] Bizzarri M., Fuso A., Dinicola S., Cucina A., Bevilacqua A. (2016). Pharmacodynamics and pharmacokinetics of inositol(s) in health and disease. Expert Opin. Drug Metab. Toxicol..

[4] Hipps P.P., Holland W.H., Sherman W.R. (1977). Interconversion of myo- and scyllo-inositol with simultaneous formation of neo-inositol by an NADP+ dependent epimerase from bovine brain. Biochem. Biophys. Res. Commun..

[5] Sun T., Heimark D.B., Nguygen T., Nadler J.L., Larner J. (2002). Both myo-inositol to chiro-inositol epimerase activities and chiro-inositol to myo-inositol ratios are decreased in tissues of GK type 2 diabetic rats compared to Wistar controls. Biochem. Biophys. Res. Commun..

[6] Su X.B., Ko A.A., Saiardi A. (2023). Regulations of myo-inositol homeostasis: Mechanisms, implications, and perspectives. Adv. Biol. Regul..

[7] York J.D. (2006). Regulation of nuclear processes by inositol polyphosphates. Biochim. Biophys. Acta BBA—Mol. Cell Biol. Lipids.

[8] Shears S.B. (2009). Diphosphoinositol Polyphosphates: Metabolic Messengers?. Mol. Pharmacol..

[9] Michell R.H. (2008). Inositol derivatives: Evolution and functions. Nat. Rev. Mol. Cell Biol..

[10] Roberts M.F. (2006). Inositol in Bacteria and Archaea. Biology of Inositols and Phosphoinositides.

[11] Deranieh R.M., Greenberg M.L. (2009). Cellular consequences of inositol depletion. Biochem. Soc. Trans..

[12] Arora M., Giuliani A., Curtin P. (2020). Biodynamic Interfaces Are Essential for Human–Environment Interactions. BioEssays.

[13] Martino A., Giuliani A., Todde V., Bizzarri M., Rizzi A. (2020). Metabolic networks classification and knowledge discovery by information granulation. Comput. Biol. Chem..

[14] Bizzarri M., Palombo A., Cucina A. (2013). Theoretical aspects of Systems Biology. Prog. Biophys. Mol. Biol..

[15] Rapoport A. (1986). General System Theory: Essential Concepts & Applications.

[16] Alberts B., Johnson A., Lewis J., Morgan D., Raff M., Roberts K., Walter P., Wilson J., Hunt T. (2017). Molecular Biology of the Cell.

[17] Küchler A., Yoshimoto M., Luginbühl S., Mavelli F., Walde P. (2016). Enzymatic reactions in confined environments. Nat. Nanotechnol..

[18] Leonard T.A., Loose M., Martens S. (2023). The membrane surface as a platform that organizes cellular and biochemical processes. Dev. Cell.

[19] Marat A.L., Haucke V. (2016). Phosphatidylinositol 3-phosphates—At the interface between cell signalling and membrane traffic. EMBO J..

[20] Balla T. (2013). Phosphoinositides: Tiny Lipids with Giant Impact on Cell Regulation. Physiol. Rev..

[21] Hokin M.R., Hokin L.E. (1953). Enzyme Secretion and the Incorporation of P32 into Phospholipides of Pancreas Slices. J. Biol. Chem..

[22] Shears S.B. (1998). The versatility of inositol phosphates as cellular signals. Biochim. Biophys. Acta BBA—Mol. Cell Biol. Lipids.

[23] Kim S., Bhandari R., Brearley C.A., Saiardi A. (2024). The inositol phosphate signalling network in physiology and disease. Trends Biochem. Sci..

[24] Wilson M.S.C., Livermore T.M., Saiardi A. (2013). Inositol pyrophosphates: Between signalling and metabolism. Biochem. J..

[25] Takenawa T., Itoh T., Fukami K. (1999). Regulation of phosphatidylinositol 4,5-bisphosphate levels and its roles in cytoskeletal re-organization and malignant transformation. Chem. Phys. Lipids.

[26] Gaspar M.L., Aregullin M.A., Jesch S.A., Henry S.A. (2006). Inositol Induces a Profound Alteration in the Pattern and Rate of Synthesis and Turnover of Membrane Lipids in Saccharomyces cerevisiae. J. Biol. Chem..

[27] Minini M., Senni A., He X., Proietti S., Liguoro D., Catizone A., Giuliani A., Mancini R., Fuso A., Cucina A. (2021). miR-125a-5p impairs the metastatic potential in breast cancer via IP6K1 targeting. Cancer Lett..

[28] Czech M.P. (2000). PIP2 and PIP3. Cell.

[29] Tóth D.J., Tóth J.T., Damouni A., Hunyady L., Várnai P. (2024). Effect of hormone-induced plasma membrane phosphatidylinositol 4,5-bisphosphate depletion on receptor endocytosis suggests the importance of local regulation in phosphoinositide signaling. Sci. Rep..

[30] Lanier L.M., Gertler F.B. (2000). Actin cytoskeleton: Thinking globally, actin’ locally. Curr. Biol..

[31] Prever L., Squillero G., Hirsch E., Gulluni F. (2024). Linking phosphoinositide function to mitosis. Cell Rep..

[32] Hille B., Dickson E.J., Kruse M., Vivas O., Suh B.-C. (2015). Phosphoinositides regulate ion channels. Biochim. Biophys. Acta BBA—Mol. Cell Biol. Lipids.

[33] Rusinova R., Hobart E.A., Koeppe R.E., Andersen O.S. (2013). Phosphoinositides alter lipid bilayer properties. J. Gen. Physiol..

[34] Suh B.-C., Hille B. (2008). PIP2Is a Necessary Cofactor for Ion Channel Function: How and Why?. Annu. Rev. Biophys..

[35] Senju Y., Lappalainen P. (2019). Regulation of actin dynamics by PI(4,5)P2 in cell migration and endocytosis. Curr. Opin. Cell Biol..

[36] Gross C. (2016). Defective phosphoinositide metabolism in autism. J. Neurosci. Res..

[37] Tariq K., Luikart B.W. (2021). Striking a balance: PIP2 and PIP3 signaling in neuronal health and disease. Explor. Neuroprot. Ther..

[38] Reversi A., Loeser E., Subramanian D., Schultz C., De Renzis S. (2014). Plasma membrane phosphoinositide balance regulates cell shape during Drosophila embryo morphogenesis. J. Cell Biol..

[39] Clarke R.J., Hossain K.R., Cao K. (2020). Physiological roles of transverse lipid asymmetry of animal membranes. Biochim. Biophys. Acta BBA—Biomembr..

[40] Wang Y.-H., Sheetz M.P. (2022). When PIP2 Meets p53: Nuclear Phosphoinositide Signaling in the DNA Damage Response. Front. Cell Dev. Biol..

[41] Divecha N., Letcher A.J., Banfic H.H., Rhee S.G., Irvine R.F. (1995). Changes in the components of a nuclear inositide cycle during differentiation in murine erythroleukaemia cells. Biochem. J..

[42] Choi S., Chen M., Cryns V.L., Anderson R.A. (2019). A nuclear phosphoinositide kinase complex regulates p53. Nat. Cell Biol..

[43] Berridge M.J. (2016). The Inositol Trisphosphate/Calcium Signaling Pathway in Health and Disease. Physiol. Rev..

[44] Putta P., Rankenberg J., Korver R.A., van Wijk R., Munnik T., Testerink C., Kooijman E.E. (2016). Phosphatidic acid binding proteins display differential binding as a function of membrane curvature stress and chemical properties. Biochim. Biophys. Acta BBA—Biomembr..

[45] Claing A. (2002). Endocytosis of G protein-coupled receptors: Roles of G protein-coupled receptor kinases and ß-arrestin proteins. Prog. Neurobiol..

[46] Tahirovic S., Bradke F. (2009). Neuronal Polarity. Cold Spring Harb. Perspect. Biol..

[47] Gassama-Diagne A., Yu W., ter Beest M., Martin-Belmonte F., Kierbel A., Engel J., Mostov K. (2006). Phosphatidylinositol-3,4,5-trisphosphate regulates the formation of the basolateral plasma membrane in epithelial cells. Nat. Cell Biol..

[48] Wang J., Richards D.A. (2012). Segregation of PIP2 and PIP3 into distinct nanoscale regions within the plasma membrane. Biol. Open.

[49] Goebbels S., Oltrogge J.H., Kemper R., Heilmann I., Bormuth I., Wolfer S., Wichert S.P., Mobius W., Liu X., Lappe-Siefke C. (2010). Elevated Phosphatidylinositol 3,4,5-Trisphosphate in Glia Triggers Cell-Autonomous Membrane Wrapping and Myelination. J. Neurosci..

[50] Li Z., Venable R.M., Rogers L.A., Murray D., Pastor R.W. (2009). Molecular Dynamics Simulations of PIP2 and PIP3 in Lipid Bilayers: Determination of Ring Orientation, and the Effects of Surface Roughness on a Poisson-Boltzmann Description. Biophys. J..

[51] Tan X., Thapa N., Choi S., Anderson R.A. (2015). Emerging roles of PtdIns(4,5)P2—Beyond the plasma membrane. J. Cell Sci..

[52] Wada T., Sasaoka T., Funaki M., Hori H., Murakami S., Ishiki M., Haruta T., Asano T., Ogawa W., Ishihara H. (2001). Overexpression of SH2-Containing Inositol Phosphatase 2 Results in Negative Regulation of Insulin-Induced Metabolic Actions in 3T3-L1 Adipocytes via Its 5′-Phosphatase Catalytic Activity. Mol. Cell. Biol..

[53] Clément S., Krause U., Desmedt F., Tanti J.-F., Behrends J., Pesesse X., Sasaki T., Penninger J., Doherty M., Malaisse W. (2001). The lipid phosphatase SHIP2 controls insulin sensitivity. Nature.

[54] Langille S.E., Patki V., Klarlund J.K., Buxton J.M., Holik J.J., Chawla A., Corvera S., Czech M.P. (1999). ADP-ribosylation Factor 6 as a Target of Guanine Nucleotide Exchange Factor GRP1. J. Biol. Chem..

[55] Siddhanta U., McIlroy J., Shah A., Zhang Y., Backer J.M. (1998). Distinct Roles for the p110α and hVPS34 Phosphatidylinositol 3′-Kinases in Vesicular Trafficking, Regulation of the Actin Cytoskeleton, and Mitogenesis. J. Cell Biol..

[56] Saarikangas J., Zhao H., Lappalainen P. (2010). Regulation of the Actin Cytoskeleton-Plasma Membrane Interplay by Phosphoinositides. Physiol. Rev..

[57] Wang F., Herzmark P., Weiner O.D., Srinivasan S., Servant G., Bourne H.R. (2002). Lipid products of PI(3)Ks maintain persistent cell polarity and directed motility in neutrophils. Nat. Cell Biol..

[58] Chen Y., Thelin W.R., Yang B., Milgram S.L., Jacobson K. (2006). Transient anchorage of cross-linked glycosyl-phosphatidylinositol–anchored proteins depends on cholesterol, Src family kinases, caveolin, and phosphoinositides. J. Cell Biol..

[59] Bretscher M.S., Aguado-Velasco C. (1998). EGF induces recycling membrane to form ruffles. Curr. Biol..

[60] Dowler S., Currie R.A., Campbell D.G., Deak M., Kular G., Downes C.P., Alessi D.R. (2000). Identification of pleckstrin-homology-domain-containing proteins with novel phosphoinositide-binding specificities. Biochem. J..

[61] Lawlor M.A., Alessi D.R. (2001). PKB/Akt. J. Cell Sci..

[62] Lang F., Cohen P. (2001). Regulation and Physiological Roles of Serum- and Glucocorticoid-Induced Protein Kinase Isoforms. Sci. STKE.

[63] Tall E.G., Spector I., Pentyala S.N., Bitter I., Rebecchi M.J. (2000). Dynamics of phosphatidylinositol 4,5-bisphosphate in actin-rich structures. Curr. Biol..

[64] Ekblad L. (2001). Localization of phosphatidylinositol 4-kinase isoenzymes in rat liver plasma membrane domains. Biochim. Biophys. Acta BBA—Mol. Cell Biol. Lipids.

[65] Watt S.A., Kular G., Fleming I.N., Downes C.P., Lucocq J.M. (2002). Subcellular localization of phosphatidylinositol 4,5-bisphosphate using the pleckstrin homology domain of phospholipase C δ1. Biochem. J..

[66] Toker A. (1998). The synthesis and cellular roles of phosphatidylinositol 4,5-bisphosphate. Curr. Opin. Cell Biol..

[67] Honda A., Nogami M., Yokozeki T., Yamazaki M., Nakamura H., Watanabe H., Kawamoto K., Nakayama K., Morris A.J., Frohman M.A. (1999). Phosphatidylinositol 4-Phosphate 5-Kinase α Is a Downstream Effector of the Small G Protein ARF6 in Membrane Ruffle Formation. Cell.

[68] Ikeda Y., Lala D.S., Luo X., Kim E., Moisan M.P., Parker K.L. (1993). Characterization of the mouse FTZ-F1 gene, which encodes a key regulator of steroid hydroxylase gene expression. Mol. Endocrinol..

[69] Blind R.D., Suzawa M., Ingraham H.A. (2012). Direct Modification and Activation of a Nuclear Receptor–PIP2 Complex by the Inositol Lipid Kinase IPMK. Sci. Signal..

[70] Vidalle M.C., Sheth B., Fazio A., Marvi M.V., Leto S., Koufi F.-D., Neri I., Casalin I., Ramazzotti G., Follo M.Y. (2023). Nuclear Phosphoinositides as Key Determinants of Nuclear Functions. Biomolecules.

[71] Czech M.P. (2003). Dynamics of Phosphoinositides in Membrane Retrieval and Insertion. Annu. Rev. Physiol..

[72] Di Paolo G., De Camilli P. (2006). Phosphoinositides in cell regulation and membrane dynamics. Nature.

[73] Overduin M., Kervin T.A. (2021). The phosphoinositide code is read by a plethora of protein domains. Expert Rev. Proteom..

[74] Fruman D.A., Chiu H., Hopkins B.D., Bagrodia S., Cantley L.C., Abraham R.T. (2017). The PI3K Pathway in Human Disease. Cell.

[75] Yoshioka K. (2021). Class II phosphatidylinositol 3-kinase isoforms in vesicular trafficking. Biochem. Soc. Trans..

[76] Burke J.E., Williams R.L. (2015). Synergy in activating class I PI3Ks. Trends Biochem. Sci..

[77] Foukas L.C., Claret M., Pearce W., Okkenhaug K., Meek S., Peskett E., Sancho S., Smith A.J.H., Withers D.J., Vanhaesebroeck B. (2006). Critical role for the p110α phosphoinositide-3-OH kinase in growth and metabolic regulation. Nature.

[78] Okkenhaug K. (2013). Signaling by the Phosphoinositide 3-Kinase Family in Immune Cells. Annu. Rev. Immunol..

[79] Samuels Y., Waldman T. (2010). Oncogenic Mutations of PIK3CA in Human Cancers. Phosphoinositide 3-Kinase in Health and Disease.

[80] Laketa V., Zarbakhsh S., Traynor-Kaplan A., MacNamara A., Subramanian D., Putyrski M., Mueller R., Nadler A., Mentel M., Saez-Rodriguez J. (2014). PIP3 Induces the Recycling of Receptor Tyrosine Kinases. Sci. Signal..

[81] Posor Y., Eichhorn-Gruenig M., Puchkov D., Schöneberg J., Ullrich A., Lampe A., Müller R., Zarbakhsh S., Gulluni F., Hirsch E. (2013). Spatiotemporal control of endocytosis by phosphatidylinositol-3,4-bisphosphate. Nature.

[82] Sopasakis V.R., Liu P., Suzuki R., Kondo T., Winnay J., Tran T.T., Asano T., Smyth G., Sajan M.P., Farese R.V. (2010). Specific Roles of the p110α Isoform of Phosphatidylinsositol 3-Kinase in Hepatic Insulin Signaling and Metabolic Regulation. Cell Metab..

[83] Guillermet-Guibert J., Bjorklof K., Salpekar A., Gonella C., Ramadani F., Bilancio A., Meek S., Smith A.J.H., Okkenhaug K., Vanhaesebroeck B. (2008). The p110β isoform of phosphoinositide 3-kinase signals downstream of G protein-coupled receptors and is functionally redundant with p110γ. Proc. Natl. Acad. Sci. USA.

[84] Yin H.L., Janmey P.A. (2003). Phosphoinositide Regulation of the Actin Cytoskeleton. Annu. Rev. Physiol..

[85] Lemmon M.A. (2003). Phosphoinositide Recognition Domains. Traffic.

[86] Singer S.J., Nicolson G.L. (1972). The fluid mosaic model of the structure of cell membranes. Science.

[87] Kervin T.A., Overduin M. (2024). Membranes are functionalized by a proteolipid code. BMC Biol..

[88] Lillemeier B.F., Pfeiffer J.R., Surviladze Z., Wilson B.S., Davis M.M. (2006). Plasma membrane-associated proteins are clustered into islands attached to the cytoskeleton. Proc. Natl. Acad. Sci. USA.

[89] Hyvönen M., Macias M.J., Nilges M., Oschkinat H., Saraste M., Wilmanns M. (1995). Structure of the binding site for inositol phosphates in a PH domain. EMBO J..

[90] Prestwich G.D. (2004). Phosphoinositide Signaling. Chem. Biol..

[91] Posor Y., Jang W., Haucke V. (2022). Phosphoinositides as membrane organizers. Nat. Rev. Mol. Cell Biol..

[92] Hilgemann D.W., Ball R. (1996). Regulation of Cardiac Na+,Ca2+ Exchange and K ATP Potassium Channels by PIP2. Science.

[93] Borreguero-Muñoz N., Fletcher G.C., Aguilar-Aragon M., Elbediwy A., Vincent-Mistiaen Z.I., Thompson B.J. (2019). The Hippo pathway integrates PI3K–Akt signals with mechanical and polarity cues to control tissue growth. PLoS Biol..

[94] Davis M.J., Wu X., Nurkiewicz T.R., Kawasaki J., Gui P., Hill M.A., Wilson E. (2002). Regulation of Ion Channels by Integrins. Cell Biochem. Biophys..

[95] Legate K.R., Takahashi S., Bonakdar N., Fabry B., Boettiger D., Zent R., Fässler R. (2011). Integrin adhesion and force coupling are independently regulated by localized PtdIns(4,5)2 synthesis. EMBO J..

[96] McNamee H.P., Ingber D.E., Schwartz M.A. (1993). Adhesion to fibronectin stimulates inositol li-pid synthesis and enhances PDGF-induced inositol lipid breakdown. J. Cell Biol..

[97] Larsson C. (2006). Protein kinase C and the regulation of the actin cytoskeleton. Cell. Signal..

[98] Niggli V. (2005). Regulation of protein activities by phosphoinositide phosphates. Annu. Rev. Cell Dev. Biol..

[99] Levina A., Fleming K.D., Burke J.E., Leonard T.A. (2022). Activation of the essential kinase PDK1 by phosphoinositide-driven trans-autophosphorylation. Nat. Commun..

[100] Krajnik A., Brazzo J.A., Vaidyanathan K., Das T., Redondo-Muñoz J., Bae Y. (2020). Phosphoinositide Signaling and Mechanotransduction in Cardiovascular Biology and Disease. Front. Cell Dev. Biol..

[101] Monti N., Dinicola S., Querqui A., Fabrizi G., Fedeli V., Gesualdi L., Catizone A., Unfer V., Bizzarri M. (2023). Myo-Inositol Reverses TGF-β1-Induced EMT in MCF-10A Non-Tumorigenic Breast Cells. Cancers.

[102] Hilgemann D.W., Feng S., Nasuhoglu C. (2001). The Complex and Intriguing Lives of PIP2 with Ion Channels and Transporters. Sci. STKE.

[103] Tong Q., Gamper N., Medina J.L., Shapiro M.S., Stockand J.D. (2004). Direct Activation of the Epithelial Na+ Channel by Phosphatidylinositol 3,4,5-Trisphosphate and Phosphatidylinositol 3,4-Bisphosphate Produced by Phosphoinositide 3-OH Kinase. J. Biol. Chem..

[104] Harraz O.F., Hill-Eubanks D., Nelson M.T. (2020). PIP2: A critical regulator of vascular ion channels hiding in plain sight. Proc. Natl. Acad. Sci. USA.

[105] Tóth B.I., Konrad M., Ghosh D., Mohr F., Halaszovich C.R., Leitner M.G., Vriens J., Oberwinkler J., Voets T. (2015). Regulation of the transient receptor potential channel TRPM3 by phosphoinositides. J. Gen. Physiol..

[106] Berridge M.J. (2009). Inositol trisphosphate and calcium signalling mechanisms. Biochim. Biophys. Acta BBA—Mol. Cell Res..

[107] Senning E.N., Collins M.D., Stratiievska A., Ufret-Vincenty C.A., Gordon S.E. (2014). Regulation of TRPV1 Ion Channel by Phosphoinositide (4,5)-Bisphosphate. J. Biol. Chem..

[108] Suh B., Hille B. (2007). Regulation of KCNQ channels by manipulation of phosphoinositides. J. Physiol..

[109] He L., Ahmad M., Perrimon N. (2019). Mechanosensitive channels and their functions in stem cell differentiation. Exp. Cell Res..

[110] Logothetis D.E., Petrou V.I., Zhang M., Mahajan R., Meng X.-Y., Adney S.K., Cui M., Baki L. (2015). Phosphoinositide Control of Membrane Protein Function: A Frontier Led by Studies on Ion Channels. Annu. Rev. Physiol..

[111] Suh B.-C., Hille B. (2005). Regulation of ion channels by phosphatidylinositol 4,5-bisphosphate. Curr. Opin. Neurobiol..

[112] Altamura C., Greco M.R., Carratù M.R., Cardone R.A., Desaphy J.-F. (2021). Emerging Roles for Ion Channels in Ovarian Cancer: Pathomechanisms and Pharmacological Treatment. Cancers.

[113] Warren P.B., ten Wolde P.R. (2005). Chemical Models of Genetic Toggle Switches. J. Phys. Chem. B.

[114] Xu C., Tobi D., Bahar I. (2003). Allosteric Changes in Protein Structure Computed by a Simple Mechanical Model: Hemoglobin T↔R2 Transition. J. Mol. Biol..

[115] Shin J., Ming G., Song H. (2015). Molecular Toggle Switch of Histone Demethylase LSD1. Mol. Cell.

